# The Gluten-Free Diet: Safety and Nutritional Quality

**DOI:** 10.3390/nu20100016

**Published:** 2010-01-14

**Authors:** Letizia Saturni, Gianna Ferretti, Tiziana Bacchetti

**Affiliations:** 1 Centro Interdipartimentale di Educazione Sanitaria e Promozione alla Salute, Ancona, 60035 Italy; Email: g.ferretti@univpm.it (G.F.); t.bacchetti@univpm.it (T.B.); 2 Dipartimento di Biochimica, Biologia e Genetica - Università Politecnica delle Marche - Ancona, 60035, Italy

**Keywords:** celiac disease, gliadin, gluten-free diet, hordein, minor cereals, pseudo-cereals, secalin, avenin

## Abstract

The prevalence of Celiac Disease (CD), an autoimmune enteropathy, characterized by chronic inflammation of the intestinal mucosa, atrophy of intestinal villi and several clinical manifestations has increased in recent years. Subjects affected by CD cannot tolerate gluten protein, a mixture of storage proteins contained in several cereals (wheat, rye, barley and derivatives). Gluten free-diet remains the cornerstone treatment for celiac patients. Therefore the absence of gluten in natural and processed foods represents a key aspect of food safety of the gluten-free diet. A promising area is the use of minor or pseudo-cereals such as amaranth, buckwheat, quinoa, sorghum and teff. The paper is focused on the new definition of gluten-free products in food label, the nutritional properties of the gluten-free cereals and their use to prevent nutritional deficiencies of celiac subjects.

## 1. Celiac Disease

Celiac disease (CD) is a chronic systemic autoimmune disorder caused by a permanent intolerance to gluten proteins in genetically susceptible individuals [1,2]. Currently CD is thought to resemble a multisystem immunological disorder rather than a disease restricted to the gastrointestinal tract [1,2]. The first accurate clinical description of CD evidenced broad flat villi and a dense chronic lymphoepithelial inflammatory cell infiltrate in the small intestinal mucosa of patients [3]. Later the progression of the abnormalities of the intestinal mucosal in response to gluten has been described in CD patients [4]. The clinical symptoms of CD differ greatly and depend on age of the patient, duration and the extent of extra-intestinal manifestations [5,6,7]. CD was thought to be a rare disease, with a prevalence of about 0.02%, however using serology and biopsy, recent studies carried out in Europe, India, South America, Australasia and USA indicate that the prevalence may be between 0.33 and 1.06% in children and between 0.18-1.2% in adults [8,9,10,11]. The highest prevalence (5.66%) in childhood has been observed in Sahrawi [12]. In other African countries, CD is rarely diagnosed, this reflects in a much lower prevalence. Population studies also indicate that a large proportion of celiac people remain undiagnosed; this is due to many clinicians being unfamiliar with the condition. 

## 2. Pathogenesis and Molecular Mechanism

**Figure 1:**
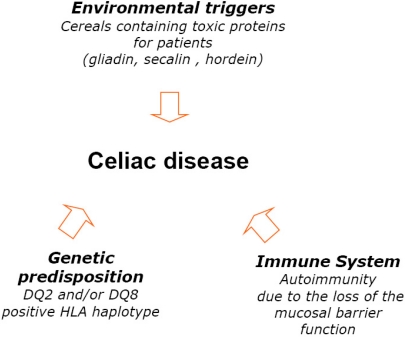
Factors involved the pathogenesis of celiac disease.

As summarized in the Figure 1, environmental, genetic and immunologic factors are involved in the pathogenesis of celiac disease [13]. Interactions between gluten peptides and intestinal epithelium [14], autoantigen (the ubiquitous enzyme tissue transglutaminase (tTG) [15] are involved and some molecular mechanisms have been hypothesized.

Gluten is a general term used to describe a mixture of wheat storage proteins (prolamins and glutenins). However other cereals have proteins that exert a toxic effect for CD patients; toxic prolamins include gliadin in wheat, secalin in rye and hordein in barley. Prolamins are characterized by a particular amino acid composition with domains with a high content of proline and glutamine. It has been demonstrated that these protein domains are resistant to degradation by gastric, pancreatic and proteases in the human intestinal brush border membrane. This results in an accumulation of relatively large peptide fragments (such as a recently described 33mer sequence) [13,14,15,16]. The study of the molecular mechanisms about the toxic effect of protein fragments has demonstrated that these peptides both *in vivo* and *in vitro* induce mucosal damage [14,16] and/or behave as “immunogenic” (*i.e.* are able to specifically stimulate HLA-DQ2 or HLA-DQ8) [17]. In fact, peptides such as the 33mer sequence, are able to stimulate intestinal immunitary cells and T cells. It has been hypothesized that one of the first events in CD pathogenesis might be a direct effect of certain wheat peptides, distinct from those recognized by T cells, on the intestinal epithelium of patients.

## 3. Gluten Free Diet

### 3.1. Nutritional State of Celiac Subjects

Previous studies have demonstrated that 20-38% of celiac patients [18,19,20,21] have some nutritional deficiencies such as calorie/protein [22], dietary fibre [23,24,25], minerals [26,27,28,29,30,31,32,33,34,35] and vitamins [27,32,36,37,38,39] (Table 1). 

Malabsorption of iron, folate, and calcium is common, as these nutrients are absorbed in the proximal small bowel. In particular, it has been reported that the frequency of iron-deficiency in celiac disease varies from 12% to 69% [36,37]. The incidence of vitamin B-12 deficiency in untreated patients ranges from 8% to 41% [36,38], though there is a relative sparing of villous atrophy in the ileum where vitamin B-12 is absorbed. Calcium, phosphorus, and vitamin D deficiencies may occur due to malabsorption or a decreased intake of milk and dairy products in an effort to avoid lactose, in fact secondary lactose intolerance resulting from decreased lactase production by the damaged villi is also common [40]. The severity of the aforementioned nutritional deficiencies is modulated by different factors: the length of time that people has lived with the active, but undiagnosed disease, the extent of damage to the gut intestinal tract and the degree of malabsorption. 

Previous studies have demonstrated that most of these nutritional deficiencies disappear after following strictly a gluten free diet [22,26,32]. Other authors have shown that gluten free diet does not guarantee adequate nutritional intake [24,25] and some nutritional deficiencies have been described after treatment with a long-term GFD for about 8-12 years (Table 1) [25,32]. The analysis of diet of 47 adults in USA showed that the recommended amount of calcium, iron and fibers was consumed only by 32, 44 and 46% of women included in the study [25]. The inadequate fiber intake is likely to be related to the composition of many GF foods made with starches and /or refined flours with low content in fiber [23,24,25]. In fact during refining, the outer layer of grain containing most of the fibre is removed, leaving only the starchy inner layer.

**Table 1:** Common nutrient deficiencies in celiac subjects at diagnosis and after Gluten Free Diet (GFD).

*At diagnosis*	*Long term GFD*
**Calorie/Protein**	Fibre
**Fibre**	
**Iron**	
**Calcium **	
**Magnesium **	
**Vitamin D **	
**Zinc**	
**Folate, Niacin, Vitamin B12, Riboflavin **	Folate, Niacin, Vitamin B12

Previous studies have investigated the nutritional composition of processed gluten-free products and it has been demonstrated that they have high levels of lipids, sugars and salt. Subjects with celiac disease tend to compensate for the restrictions of a gluten-free diet by eating foods containing high levels of fat, sugar and calories, therefore celiac patients may show an excessive consumption of total fats and saturated fats [41]. Mariani *et al.* [41] reported that diet of CD adolescent patients was hyperproteic and hyperlipidic and contained low amounts of carbohydrates, iron, calcium, and fiber. Polito *et al.* [42] and Rea *et al.* [43] observed an excess in energy, animal protein, and lipid intake, which was partially responsible for the high percentage of overweight patients. As far as concerns fat composition of gluten-free products, it has been evidenced that they contain *trans* fatty acids [44] that may provoke metabolic imbalance when in association with a inadequate intake of essential fatty acids [45]. All these components have a negative effect on health and this should be seriously taken in account because the limited choice of food products in the diet of celiac induces a high consume of packaged gluten-free products, such as snacks and biscuits [44]. A high intake of dietary lipids is a major factor influencing the development of diseases such as coronary heart disease and obesity [46]. Therefore, it has been suggested that paradoxically, a strict gluten-free diet may be a nutritional risk factor for CD patients, because it leads these subjects to incorrect alimentary choices [41].

### 3.2. Safety and Nutritional Aspects of Gluten Free-Diet

It has to be stressed that there are many aspects related to the food safety such as contamination by biological (micro-organisms such as bacteria, viruses, parasites, moulds), by physical factors or chemical substances (acrylamide, PCBs and dioxins, persistent organic pollutants). The contamination of foods may occur also through environmental toxins (heavy metals, PCBs, and dioxins), or through the intentional use of various chemicals, such as pesticides, animal drugs, and other agrichemicals. All these factors may pose significant health risks to consumers, including celiac subjects. In the present study, the term “safety” was referred mainly to the naturally gluten-free foods or foods specially processed to reduce gluten content. Since the discovery that wheat was a key environmental factor that triggered celiac disease in susceptible individuals, the relationship between the disease and the ingestion of wheat gluten proteins has became an essential part of the definition of “Gluten Free Diet”. GFD means that CD subjects have to exclude all foods and medications containing gluten. 

As summarized in Table 2, cereals containing gluten (wheat, rye, barley) and hybrids such as Khorosan wheat (Kamut®), spelt (sometimes called *Farro*) and triticale (a combination of wheat and rye) are not allowed. Other products such as semolina (durum wheat), einkorn, bulgur and wheat derivatives (wheat germ, wheat bran, whole wheat and cracked wheat) have also to be avoided. Additionally all foods derived from gluten-containing cereals including pasta, breads and crackers are not allowed. Malt is also toxic for CD subjects because it is a partial hydrolysate of barley prolamins [47]. Therefore barley malt, malt syrup, malt extract, malt flavorings cannot be inserted in CD diet. Beer usually contains appreciable quantities of hordein (barley gluten prolamin) [47], however some low gluten beer has been presented in the market. 

For several years oat have been excluded from the CD diet because it was thought that avenin (the storage protein found in oats) was also toxic to CD patients. Moreover the use of oats in GFD is still debated in relation to a possible cross-contamination with gluten-containing grains, as demonstrated in some commercial products in the United States. However some studies have demonstrated gluten contamination either in naturally gluten free products (soybean, rice, millet, corn, buckwheat) or in industrially-purified gluten free flours [49]. As far as concern oat, recent studies have revealed that when consumed in moderation, oat free from cross-contamination with gluten-containing grains, is well-tolerated by most children and adults with CD even in long-term use [48,50,51,52,53]. The literature also suggests that pure oats can be beneficial to those individuals with CD who tolerate it, and its palatability may help to increase compliance with a gluten-free diet [54] and improve the nutritional value [55,56,57]. 

**Table 2:** Vegetable and animal foods allowed or not allowed in gluten-free diet.

ALLOWED	NOT ALLOWED
Vegetable foods	
**Cereals **	**Cereals **
Corn	Wheats (Spelt ,semolina, durum)
Rice	Rye
Sorghum	Barley
Oat *	Triticale
**Minor cereals**	Kamut®
Fonio	Malt
Teff	
Millet	
Teosinte	
Job’s tears	
**Pseudo-cereals**	
Buckwheat	
Quinoa	
Amaranth	
**Vegetables**	
**Fruits**	
**Pulses**	
**Nuts **	
**Other plant foods **	
Tapioca	
Soybean	
Potato	
Root crops	
**Vegetable oils**	
**Animal foods**	
**Milk and derivatives**	
**Plain meat**	
**Fish**	
**Egg**	
**Butter**	

* oat, oat bran and oat syrup could be allowed in the GFD only if they are recommended by patient’s healthcare team

As mentioned, several cereals containing gluten have to be avoided, however a lot of other vegetable and animal foods such as fish, poultry and meats, as well as fruits and vegetables, are permitted in their natural state. Rice, corn and potatoes have been widely used as substitute of gluten-containing grains. A number of nutrient dense grains, seeds, pulses offer increased variety, improved palatability and high nutritional quality for the GFD (Table 2) [54,59,61]. Minor cereals - so called because they are less common and are only grown in a few small regions of the world- such as fonio, teff, millet, teosinte and Job’s tears, and pseudo-cereals producing small grain-like seeds (buckwheat, Tartarian buckwheat, quinoa and amaranth grain) are allowed (Table 3). Minor cereals and pseudo-cereals may be sold whole, milled into flour, flakes or grits, puffed and /or incorporated into pasta, cereals, crackers and other gluten-free specialty products. In the last years, the range of processed gluten-free products is increased: breads, biscuits, and pasta produced by gluten-free ingredients and food additives have been proposed on the market. Therefore, celiac patients need to pay attention to food labels, looking for words such as wheat starch, wheat bran, graham flour, kamut® and hydrolyzed wheat protein. 

**Table 3:** Characteristics of some plant (pseudo-cereals and minor cereals) gluten-free alternative.

*Teff*	*Teff* is a grass native to Ethiopia, that belongs to the Poaceae family. It is also grown in India, Australia and Northwestern USA. Teff is the smallest of all grains in the world (about 100-150 teff grains equal the size if 1 wheat kernel) and its grains range from milky white to almost black. Teff has a unique nutty, mild molasses-like flavour and is sold as whole grain and as flour. In Ethiopia flour is fermented 1-3 days to make “Injera”, a sour-dough-type flat bread .
*Fonio*	*Fonio* is a typically cereal in Sudan or Ethiopia where it is considered to be the tastiest of all cereals
*Millet*	The term *millet* refers to various grasses that grown in semi-arid regions of the world. There are a lot of species, but only pearl and finger millets are used for food consumption. Millet is closely related to corn and belongs to Gramineae family. Milled seeds are very small and can be yellow, white, gray or red. It is similar in texture to rice flour.
*Amaranth*	*Amaranth* is a broad-leafed plant which produces florets containing thousands of tiny grain-like, tan-colored seeds. Although it is used as grain, it is not an actual grain but a member of the Amaranthaceaea family. Amaranth seeds have been used as a staple by many ancient civilizations around the word. It has a robust nutty flavour.
*Buckwheat*	*Buckwheat* is thought to have originated in China. The larger producers of buckwheat today are China, Japan, Russia and North America, although it is also grown in Europe, India, Australia. Buckwheat is botanically classified as a fruit, not a cereal grain and is of the Polygonaceae family, which is closely related to rhubarb. It is triangular in shape and has a black shell. The outer shell is removed and the kernel inside is known as a great.
*Quinoa*	*Quinoa* has been consumed for thousands of years in South America and was a staple of the Incas. It is not actually a grain but the seeds of a broad-leafed plant from the Chenopodiaceae family which is a close relative of the weed, lamb’s quarters. There are hundreds of varieties of quinoa, ranging in colour from white to red and purple to black.

### 3.3. Food Labelling and Legislative Aspects

The absence of gluten in natural and processed foods represents a key aspect of food safety of the gluten free diet. The term “gluten free” was introduced in food labels several years ago. The current Codex Standard for GF foods was adopted by the Codex Alimentarius Commission of the World Health Organization (WHO) and by the Food and Agricultural Organization (FAO) in 1976 and amended in 1983 (Codex Alimentarius Commission, 1983). The definition came under review in the 1990s. 

It has to be stressed that gluten contamination in processed GF products cannot be totally avoided. There is no agreement about the amount of dietary gluten that CD subjects may introduce without damaging the mucosa of the small intestine. Some authors consider safe a threshold for which gluten contamination should be set: less than 30 mg of gluten per day [62]; between 10 and 100 mg intake daily [63]; higher than 50 mg per day [64]. 

In USA, Food and Drug Administration (Codex Standard, 2007) is proposing to define the term gluten-free to mean that a food bearing this claim does not contain: an ingredient that is not allowed (wheat, rye, barley or their crossbred hybrids); an ingredient that is derived from a prohibited grain and that has not been processed to remove gluten; an ingredient that is derived from a prohibited grain that has been processed to remove gluten, if the use of that ingredient results in the presence of 20 ppm or more gluten in the food and, at last, 20 ppm or more gluten.

Recently, European countries have accepted the definition of GF designed by Codex Alimentarius (the gluten free certification organization, 2007) [65]. The term 'gluten-free' will refer only to foods containing less than 20 ppm of gluten (Table 4). In addition, the claim 'very low gluten' will be used for foods such as bread, produced using cereals that have been specially processed to remove most of the gluten and containing gluten less than 30 mg daily. Manufacturers can use the new labeling system immediately; however, they have time to adapt to the new rules until 1 January 2012. 

We may expect that new type of labeling will help CD subjects to make safer and more informed food choices.

**Table 4:** New Codex Alimentarious Standards for Gluten-Free foods.

**- Gluten-free foods are dietary foods **
a) consisting of or made only from one or more ingredients which do not contain wheat (*i.e.*, all Triticum species, such as durum wheat, spelt and kamut*®*), rye, barley, oats^1^ or their crossbred varieties, and the gluten level does not exceed 20 mg/kg in total, based on the food as sold or distributed to the consumer,
and/or
b) consisting of one or more ingredients from wheat (*i.e.*, all Triticum species, such as durum wheat, spelt, and kamut*®*), rye, barley, oats^1^ or their crossbred varieties, which have been specially processed to remove gluten, and the gluten level does not exceed 20 mg/kg in total, based on the food as sold or distributed to the consumer.
**- Foods specially processed to reduce gluten content to a level above 20 up to 100 mg/kg **
These foods consist of one or more ingredients from wheat (*i.e.*, all Triticum species, such as durum wheat, spelt, and kamut*®*), rye, barley, oats^1^ or their crossbred varieties, which have been specially processed to reduce the gluten content to a level above 20 up to 100 mg/kg in total, based on the food as sold or distributed to the consumer.
^1 ^Oats can be tolerated by most but not all people who are intolerant to gluten. Therefore, the allowance of oats that are not contaminated with wheat, rye or barley in foods covered by this standard may be determined at the national level.

### 3.4. General Dietary Advices for Celiac Subjects

Even if studies related to nutritional state of CD are limited for duration and study design, all demonstrated that nutritional therapy is an essential part of a management of CD.

Different countries show different lifestyles and nutritional habits; therefore is not easy to suggest dietary recommendations that could be applied to celiac people of different ethnic groups. Here we describe some dietary advices and the nutritional properties of some foods that can be included in the gluten-free diet to prevent malnutrition.

*Carbohydrate.* Complex and simple carbohydrates intake should represent about 55% of total calories. As aforementioned, even if the grain sources of carbohydrate are limited in the GFD, legumes and a wide variety of grains and seeds are permitted (Table 2). In the last years, the nutrient composition of minor cereals and pseudo-cereals has been characterized and it has been demonstrated that they represent a good source of carbohydrate (Table 5), dietary fibre, minerals, vitamins and phenols [66,67,68,69,70,71,72].

**Table 5:** Chemical composition (% dry mass) of Amaranth, Quinoa, Oat and Buckwheat compared to Wheat.

Component	Amaranth	Quinoa	Buckwheat	Oat	Wheat
**Starch**	67.3	69.0	67.2	**nd**	61.0
**Protein**	15.2	13.3	10.9	**13**	11.7
**Fat**	8.0	7.5	2.7	**7.5**	2.0
**Minerals**	3.2	2.6	1.59	**3.1**	1.8

*Dietary fibre.* Dietary fibre is a complex mixture of plant materials and molecules that are resistant to breakdown (digestion). Some fibre components exert physiological roles and are metabolized by gut bacteria. Several studies have demonstrated that high-fibre diets prevent many human diseases, colon cancer, coronary heart disease and diabetes [73]. An adequate intake (20-35g/d) of fibre has to be recommended in CD subjects. As aforementioned, some studies have reported that GFD is associated with a lower intake of dietary fibre [23,24]. As summarized in Table 6, the fibre content in minor cereals and pseudo-cereals range from 7 to 10 g/100 g [68]. These levels are higher with respect to other plant foods fruits, nuts, pulses and cereals such as corn and rice. Therefore, their use in GDF can help to increase fibre intake in CD patients. 

**Table 6:** Fibre content in different plant foods.

	Fibre (g/100g)
**Cereals**	
Oat	10.3
Wheat	9.5
Barley	9.2
Teff	8
Corn	7.3
Spelt	6.8
Rice	2.8
**Pseudo-cereals **	
Buckwheat	10
Quinoa	7
Amaranth	6.7
**Fruit and vegetable **	0.5-5.0
**Nuts **	4.0-12.0
**Pulses**	5.0-18.0

*Protein.* Dietary protein intake should represent about 15% of total calories. In GDF the main dietary source of protein are animal foods such as meat, milk and dairy products, eggs and fish. Plant foods which are useful sources of protein include legumes, nuts, seeds and gluten free cereals. In the last years, the protein content of pseudo-cereals and minor cereals has been investigated and it has been demonstrated that is higher with respect to wheat (Table 5), moreover the quality of the protein is much better [74,75]. In particular lysine, the limiting amino acid in cereals can be found in high amounts. The high content of arginine and histidine, both essential for infants and children, makes Amaranth and Quinoa interesting for the nutrition of CD children. Moreover pseudo-cereals and minor cereals contain amino acids like methionine and cysteine which are essential to human health. Protein quality not only depends on the amino acid composition, but also on the bioavailability or digestibility. Protein digestibility, available lysine, net protein utilisation (NPU) or protein efficiency ratio (PER) are widely used as indicators for the nutritional quality of proteins. In this respect, the values for pseudo-cereal proteins are definitively higher when compared to cereals and are close to those of casein [74,75]. 

*Lipids.* Total fat intake should represent about 25-30% or less of total calories. The intake of unsaturated fat (monounsaturated and polyunsaturated) should be preferred. Monounsaturated and polyunsaturated fatty acids should provide more than 15% and 10% of total calories, respectively (50% and 25% of total fat) (Table 4). In fact, monounsaturated fats and omega-3 fatty acids intake has been associated with reduced cardiovascular disease risk [76]. They are found in foods such as vegetable oils, nuts, seeds and higher fat fish including salmon, trout and herring. On the contrary saturated fatty acids, manly found in animal foods (meat, poultry, whole milk dairy products) and in tropical oils, should be limited (8-10% of total calories). Also *trans* fatty acids play a negative effect on development of atherosclerosis [45], therefore their intake should be limited at less than 1% of total calories (about 5g/die). As previously described the levels of saturated and *trans* fatty acids in processed gluten free products are higher with respect to conventional foods [44]. Therefore, it is important that celiac subjects pay attention to the food label and to hydrogenated fats content. 

Although the lipid content of pseudo-cereals is higher compared to other plant foods (Table 5), they are characterised by a higher content of unsaturated fatty acids in particularly of linolenic acid [71,77], an omega-3 fatty acid essential for all mammals. A consumption of α-linolenic acid (2 to 3 g per day) has been considered important for the primary and secondary prevention of coronary heart disease [78]. Amaranth contains a high amount of squalene, a highly unsaturated open chain triterpen, which is usually only found in liver of deep see fish and other maritime species [71,77].

*Micronutrients*. To avoid micronutrient deficiencies in celiac subjects, the amount of fruits and vegetables should be increased. An intake of at least five portions of fruit or vegetable a day has been recommended to help to reduce the risk of some diseases (cancer, heart disease). Fruit and vegetables are low in energy and rich in vitamins and minerals. Moreover, they contain phytochemicals and antioxidant compounds that exert a protective effect against diseases associated with oxidative damage [79,80]. 

*Mineral salts. *These are essential nutrients. They include major minerals (calcium, phosphorus, sodium, potassium, chloride and magnesium) and trace elements (iron, zinc, selenium). In addition to animal products, also vegetable foods contain a significant amount of minerals. The total content of minerals in amaranth, quinoa and oats is about twice as high as in other cereals (Table 4) [77,81,82]. In teff, iron and calcium contents (11-33 mg and 100-150 mg, respectively) are higher than those of wheat, barley, or sorghum and rice. In Ethiopia, an absence of anaemia seems to correlate with the levels of teff consumption and is presumed to be due to the grain's high content of iron. The content of minerals in buckwheat seeds is lower than in wheat. However, except for calcium, buckwheat is a richer source of nutritionally important minerals than many cereals such as rice, sorghum, millet and maize [77,81]. Minerals are located in the germ; therefore, we may expect that they are not completely lost during the refining process. 

*Vitamins*. An adequate intake of vitamins is particularly important for celiac patients to prevent vitamin deficiencies. Folic acid is present in green leafy vegetable, liver and cereals. High concentration of folic acid has been found in gluten free cereals such as quinoa (78.1 μg/100 g) and amaranth (102 μg/100g) with respect to wheat (40 μg/100 g). Both amaranth, quinoa and oats are also good sources of riboflavin, vitamin C and vitamin E [72,82,83]. The vitamins B2 and B6 are also present in buckwheat seeds [83]. Finally, it should be noted that a growing number of gluten-free specialty products are now being fortified with vitamins and minerals. 

**Table 7:** Phenolic acid content in different plant foods and derivatives.

Sample	Phenolic acid (mg/100g)
**Whole Grains:**	
Foxtail millet	390.7
Pearl millet	147.8
Rye	136.2
Wheat	134.2
Barley	45.0-134.6
Sorghum	38.5-74.6
Finger millet	61.2
Maize	60.1
Oat	47.2
Rice	19.7-37.6

**Brans**	
Wheat	452.7
Rye	419
Oat	65.1

**Fruit and beverages**	
Coffee	97
Blueberry	85
Green and black teas	30-36
Dark plum	28


*Phytochemicals.* All plant-derived foods contain phytochemicals such as polyphenols which affect their organoleptic and nutritional properties. Awareness of their importance in human nutrition has been aroused because of their potential beneficial effects on human health (in fact they are able to reduce risk of cardiovascular disease, ischemic stroke, type II diabetes, metabolic syndrome and gastrointestinal cancers [73]. Moreover they have been reported to have antiviral, anti-allergic, antiplatelet, anti-inflammatory, antitumor and antioxidant activities [79,80]. As previously reported, the main food sources of these compounds are fruits, vegetables, wine, teas [79,80]. However, more recently it has been reported that cereals and pseudo-cereals could represent a good source of polyphenols. The levels of total phenolic acids in some cereals and gluten-free products are summarized in table 7 [85,86,87]. Flavonoids are polyphenolic compounds (flavonols, flavones, flavanones, isoflavones, catechins, anthocyanidins and chalcones) occurring in fruits, vegetables and beverages (tea, coffee, beer, wine and fruit drinks). They contribute to the colour of several fruits and vegetables. Flavonoids have been reported also in the pericarp of pigmented varieties of barley, maize, rice, rye and wheat [83]. The levels of antocyanins in different fruits and pigmented cereal grains are summarized in Table 8 [88,89]. Other flavonoids mainly found in fruits and vegetables are also reported in cereals. For example the flavone apigenin, a typical compound found in parsley and celery, is also reported in millet, oats and sorghum. Flavonones, have been quantified in sorghum and oats [85,86,87].

**Table 8:** Anthocyanin content of different fruits and pigmented cereal grains (mg/100 g).

Sample	Anthocyanin (mg/100g)
Blue barley	0.4
Pink maize	9.3
Red maize	56
Blue maize	22.5
Purple maize	96.5
Black rice	22.8-32.7
Black sorghum	94.4
Corn shaman blue	32.7
Blue wheat	10.6-21
White wheat	0.7
Purple wheat	1.3-13.9
Blackberry cultivars	131 to 256

Several phytochemicals behave as antioxidants *in vitro*. Recently the total antioxidant capacity of gluten free cereals has been studied. The results have shown that buckwheat and quinoa possess the highest antioxidant potential among the studied cereals and pseudo-cereals [85,86,87,90].

## 4. Conclusions

The prevalence of celiac disease is increased in the last years. Previous studies have demonstrated that gluten free diet products are poor sources of minerals (such as iron), vitamins (such as folate, thiamine niacin and riboflavin) and fibre [23,24], therefore the nutritional content of gluten-free foods is an increasing area of concern. A promising area is the use of minor or pseudo-cereals such as amaranth, buckwheat, quinoa, sorghum and teff. Minor cereals and pseudo-cereals may be used to prepare several gluten-free specialty products. The technological and nutritional properties of these alternative cereals as wheat replacements have been investigated and it has been suggested that their use could improve intakes of protein, iron, calcium and fibre of celiac patients [54]

In a systematic review of literature on oat inclusion in the gluten-free diet, Størstud *et al.* [55] noted that the inclusion of oats would increase the variety and nutrient content of the gluten-free diet. Other authors have confirmed that oats exert a positive effect on GDF [56]. Recently, Lee *et al.* [54] demonstrated that the adding of three servings of gluten-free alternative grains, including oats and quinoa, positively impacts the nutrient profile (fibre, thiamine, riboflavin, niacin, folate and iron) of the grain portion of the gluten-free diet. Minor cereals and pseudo-cereals are important sources of proteins and carbohydrates, especially fibre, and would permit a wider choice of foods for celiac individuals when selecting foods.

As far as concerns food price, Stevens *et al*. [92] have reported previously that commercially available products labelled gluten-free were significantly more expensive than comparable products. Lee *et al.* [54] have suggested that the alternative grains such as oats and quinoa could represent less expensive alternative with respect to standard gluten-free diet choices, quinoa and oats could increase dietary compliance also by reducing the economic burden of the diet. However further studies are necessary to better investigate whether the use of pseudo-cereals and minor cereals can contribute to reduce nutritional deficiencies of treated CD subjects and decrease prices of gluten free foods.
